# Assessing Knowledge of HIV Vaccines and Biomedical Prevention Methods Among Transgender Women in the New York City Tri-State Area

**DOI:** 10.1089/trgh.2019.0049

**Published:** 2020-06-08

**Authors:** Nicholas Kenji Taylor, Maria R. Young, Van Don Williams, Jorge Benitez, DaShawn Usher, Scott M. Hammer, Hong-Van Tieu, Magdalena E. Sobieszczyk

**Affiliations:** ^1^ Department of Medicine, Division of Infectious Diseases, Columbia University College of Physicians and Surgeons, New York, New York, USA.; ^2^ Department of Family and Community Medicine, University of California San Francisco, San Francisco, California, USA.; ^3^ New York Blood Center, New York, New York, USA.

**Keywords:** health disparities, HIV/AIDS, MSM, prevention, transgender/transsexual vaccine

## Abstract

**Purpose:** To lower the HIV risk of transgender women, it is imperative to understand their unique HIV prevention needs and design biomedical prevention interventions that are responsive to the psychosocial, behavioral, and clinical needs of these communities. Preventive HIV vaccines are an important modality under investigation in diverse study participants. We sought to assess the knowledge of HIV vaccine research and the most common barriers and facilitators to participation in HIV vaccine studies among HIV-negative transgender women living in New York City.

**Methods:** Six focus groups were conducted among 29 participants recruited in the New York City tri-state area from December 2014 to July 2015. Prefocus group quantitative questionnaire assessed demographic, behavioral information, knowledge of preventive vaccine research, and reasons for potential participation in prevention studies.

**Results:** Median age of participants was 29 years and 41.4% identified as white. Over half of participants have heard of preventive vaccine research and majority indicated that an important factor in participating in HIV prevention research is to help the community collective effort. Key barriers that emerged were fear of side effects, feelings of exclusion from biomedical research. Facilitators to participation in prevention studies included trusting relationships with providers.

**Conclusions:** These barriers and facilitators are important to consider in the design of studies inclusive of trans communities and transgender-specific prevention strategies. Barriers may be overcome by disseminating accurate information via social media or health providers.

## Introduction

Over 1.1 million people are living with HIV in the United States today and it is estimated that this number grows by 50,000 each year.^1^ Transgender women (male-to-female transgender persons) are a diverse group bearing a significant burden of HIV disease and represent a key population at risk.^2^ A systematic review revealed that self-identified transgender women have high rates of confirmed HIV infection (27.7%), as high as 56.3% for African American transgender women, and 68.0% for transgender women sex workers.^2–4^ These estimates exceed prevalence estimates in men who have sex with men (MSM, 25.0%), a community commonly regarded as the highest risk group.^2,5^ In New York City, earlier data reveal that 49.6% of Latina and 48.1% of African American transgender women are HIV-positive, and more recently, the cumulative incidence of HIV among transgender women has been shown to be 2.8%.^6,7^

The reasons for increased HIV risk in transgender women are unique to these communities and have been associated with receptive anal sex, psychosocial factors such as stigma and bias along with structural factors such as employment, housing, and economic instability.^8^ These psychosocial factors can lead to depression, risk-taking behaviors, and poor access to health care and health education.^8^ Additionally, transgender women face significant barriers to accessing routine health services, in particular HIV testing services, and HIV treatment that may negatively impact health outcomes.^8^ One study in San Francisco with the largest sample of transgender individuals to date (291) found higher HIV-related mortality and viral load in HIV-infected transgender women compared to cisgender individuals.^9^ And thus, it is imperative to consider the unique psychosocial, behavioral, and clinical context of prevention needs among transgender women to design studies and interventions that are relevant to these communites.^10,11^

For these reasons, inclusion of transgender women in HIV vaccine and biomedical prevention research is critically important. Yet efforts have not focused explicitly on engaging and recruiting this population and transgender women have been underrepresented in biomedical HIV prevention studies.^10–14^ Moreover, very limited data exist on the barriers and facilitators to knowledge of and participation in prevention studies and only one addresses participation in preventive HIV vaccine trials.^15–18^

To address these gaps, we conducted a series of focus groups among transgender women in the New York City tri-state area (NY, NJ, and CT) to evaluate knowledge of HIV prevention and to assess the barriers and facilitators to participation in HIV vaccine clinical trials among HIV-negative transgender women. The participants also advised on the development of community engagement materials that are inclusive of the transgender community.

## Methods

### Recruitment of participants

Participants were recruited using online and social media venues and utilizing partnerships with community based organizations (CBO). Ads were placed on Facebook and Craigslist. Additionally, the research team identified CBOs throughout New York City that deliver services to transgender individuals; research staff visited their locations to distribute flyers and disseminate information about the focus groups. Interested individuals completed a phone or online screening questionnaire to determine eligibility for participation in a focus group. Eligible participants were contacted to schedule a focus group, and were given date options either during the day or in the evening. Focus groups were held at CBOs and Columbia University Medical Center. We obtained written informed consent in-person before the start of each focus group. Compensation included a meal, $40 USD, and a two-way NYC MTA Metrocard to cover transportation for those participants using the MTA subway. The study was approved by the Institutional Review Boards at Columbia University and the New York Blood Center.

Eighty-eight individuals were screened between December 1, 2014 and July 9, 2015 with 29 (32.9%) ultimately participating in six focus groups during that time period. Fifty-three provided contact information and indicated willingness to be contacted; of those, the most common reasons for ineligibility were being cisfemale or MSM.

### Description of study population

Participants were eligible to participate in the study if they self-reported being HIV-negative, were between 18 and 55 years old, reported being assigned male at birth and currently self-identified as either female or as a transgender woman, and resided in New York City or the tri-state area. Participants were excluded only if they used injection drugs in the past 12 months or known to be HIV-positive.

### Focus group procedures

The focus groups evaluated the following domains: (1) knowledge of HIV vaccines and biomedical prevention methods; (2) awareness of HIV vaccine trials; (3) barriers to participation in HIV vaccine trials; and (4) feedback on educational and recruitment materials targeted at transgender women.

The focus group guide contained semi-structured open-ended questions and focus groups were led by two trained facilitators. All focus groups were digitally recorded and transcribed. Every focus group included a 15-min educational presentation on biomedical HIV prevention that covered pre-exposure and post-exposure prophylaxis and HIV vaccines and was conducted after the main discussion. Furthermore, recruitment and educational materials targeting transgender women were developed as a result of feedback received during the first four focus groups and were presented at the last two groups for further refinement.

### Measures

Before the start of focus groups, participants completed a paper-based 25-question survey about demographic characteristics, risk behaviors related to sex behavior and substance use, and and facilitators to participation in HIV prevention trials. The questionnaire was created using best practices from standardized data collection tools. Questions were multiple choice with optional free-response available. To assess risk behaviors, participants were asked to list their number of sex partners in the past 12 months, as well as the gender identify of their partners, substance abuse habits of their partners, and HIV infection status of their partners. Participants were additionally asked to report on their own drug use, incidence of sexually transmitted infections in the past 12 months, and behaviors around sex that is, exchanging sexual acts for money or gifs.

### Data analysis

Two researchers (K.T. and M.Y.) independently reviewed and conducted *in vivo* coding of all transcripts after study completion. The two researchers then met to discuss and generate any new codes through an iterative process and resolved any lingering coding differences through consensus and cross reference with notes, memos, and quantitative data. Using these codes, the researchers then together developed themes from these codes. Finally, themes were reviewed by the full investigation team closely familiar with the transgender community.

## Results

### Participant characteristics

Eighty-eight individuals were screened with 29 (32.9%) ultimately participating in 6 focus groups. There were a median of 5 (range 4–6) participants in each focus group and their median age was 29 years (IQR of 20–60). Twelve (41.4%) participants were White/non-Hispanic, 8 (27.6%) were Hispanic, 5 (17.2%) were Black, 3 (10.3%) Asian/Pacific Islander, 3 (10.3%) of mixed race, and 1 (3.4%) Native American. Eleven (37.9%) reported part-time work and 9 (31.0%) were unemployed and looking for employment. Nine (31.0%) completed college or higher education. Three (10.3%) selected “Other” when asked about their gender identity and listed “gender queer, female, femme, trans,” “girl,” and “transsexual male-to-female (MTF)” in their response. Eight (27.5%) selected “Other” when asked about their sexual identity; among these, 4 (13.7%) open-text responses included “queer,” “parasexual,” “polysexual,” and “strictly I'm a woman who loves only men.” Twenty (68.9%) had heard about HIV vaccine studies before participation in the focus group. The majority of participants were recruited for the focus groups through internet ads (41.4%) or through referral from a friend/other participants (37.9%). Other transgender women were recruited from in-person outreach (17.2%) and from a flyer/poster or HIV vaccine research site referral (3.4%).

Participants were asked about their risk behaviors in the previous 6 months. Twelve (41.4%) had sexual intercourse (vaginal, anal, and oral) with a man and 11 (37.9%) with women. Eight (30.7%) individuals reported having sexual intercourse with two or more male sexual partners. Six (18.5%) individuals reported having sexual intercourse with two or more female sexual partners. One (3.7%) reported having sex with an HIV-infected partner and one (3.7%) reported having sex with a partner who injects drugs in the past 12 months. Two (6.9%) received money, drugs, gifts, or services in exchange for sex in the past 12 months. Finally, two (7.4%) had a sexual transmitted infection and two (7.4%) used cocaine in the past 12 months.

### Facilitators to participation

Participants identified the following facilitators to participation in HIV vaccine trials: connections and information via social networks, trusting relationships with providers experienced in transgender care, and monetary nonmonetary incentives.

### Connections and information via social networks

Sixteen (55.2%) transgender women had previously heard about HIV vaccine studies from the internet. Participants cited online recruitment, education, vaccine information, and social networks as factors facilitating participation in HIV vaccine research. Sites frequented by participants included Facebook, Craigslist, Reddit, Tumblr, and Twitter. Reasons for use of social media included ease of access to larger numbers of transgender women, 24-h availability, and most importantly, confidentiality. Participants noted a stronger connection to the online transgender community beyond the tri-state NYC area more so than in-person relationships, as summarized by one participant:

I communicate with more transgenders on an online basis, like as in Facebook…but I don't have a public relationship with transgenders.

### Recommendation from a provider experienced in transgender care

Transgender women identified health care providers both as gatekeepers and roadblocks to accessing medical care. Participants reported that providers frequently ignored name and pronoun preferences or were uncomfortable interacting with the LGBTQ community. These powerful relationships were often stressful as one participant noted:

Since I started seeing my physician for my hormone replacement, I was sent to an ophthalmologist and…my doctor sent over the alias to call me by, and they totally ignored it… And it truly puts the individual under a lot of stress, because it's our self-identity that is ours, and we don't want anyone abusing that.

However, 79.3% of the participants said they had a provider or clinician with whom they had a trusting relationship. In both the surveys and focus groups, participants consistently characterized these providers as respectful, empathic, confidential, knowledgeable about transgender issues, inclusive, and patient-centered in their care. As one participant noted:

My health care provider is a specialist in transgender health services and I have sought my doctor out in the past for medical referrals for non-trans health-related issues.

While transgender women described both negative and positive experiences with providers, they regularly identified a trusted doctor or clinic as a source of more information and counseling if they were thinking about joining an HIV vaccine or biomedical prevention trial.

### Monetary and nonmonetary assistance

Eighty-two percent of participants said in the prefocus group survey that they were drawn to participate in study trials to help the community collective effort. Furthermore, empowering messaging such as “Stand Up for your community,” “Be a Voice,” and “What are you proud of?” were most popular with participants. At the same time, they identified several other monetary or access to service-related factors that would make participation more likely (Table 1). Grocery vouchers, condoms, dental dams, child care, access to HIV testing and counseling and medical care were all identified by participants from a list of options in a prefocus group survey as facilitators of their participation in research. In the focus groups, several participants described difficulty finding steady jobs and therefore meeting basic needs, precluding their participation in research. The same themes emerged during the focus group discussions. Simply put, one woman said:

**Table 1.** Factors Associated with Willingness to Participate in HIV Prevention Studies

Question: What would make your participation more likely? (N=29) Likert-scale responses					
	Least likely				Most likely
	1	2	3	4	5
Cash compensation for your time	0.0%	13.3%	3.3%	20.0%	63.3%
Knowing participating in a study might help me stay HIV-negative	0.0%	12.5%	8.3%	20.8%	58.3%
Knowing participating in a study give me a way to find out about my health on an ongoing basis	8.3%	8.3%	12.5%	37.5%	33.3%
Knowing participating in a study helps my community	4.2%	0.0%	16.7%	25.0%	54.2%
Child care or reimbursement for cost of child care	37.5%	12.5%	8.3%	8.3%	33.3%
Confidentiality regarding my participation	3.8%	0.0%	34.6%	23.1%	38.5%
Food or grocery vouchers	7.7%	7.7%	23.1%	23.1%	38.5%
Condoms, dental dams	23.1%	7.7%	26.9%	11.5%	30.8%
Knowing participating in a study I would receive ongoing risk HIV reduction counseling	4.3%	13.0%	17.4%	34.8%	30.4%
Referrals for other community services	12.0%	16.0%	20.0%	20.0%	32.0%
Public transportation vouchers	4.0%	0.0%	32.0%	36.0%	28.0%
Hygiene kits	26.9%	19.2%	23.1%	7.7%	23.1%
Clothing	24.0%	20.0%	24.0%	4.0%	28.0%
Gift cards	7.4%	14.8%	14.8%	22.2%	40.7%
Transportation	11.5%	3.8%	34.6%	30.8%	19.2%
Morning appointments	34.6%	30.8%	23.1%	7.7%	3.8%
After work appointments in the evening	8.0%	4.0%	24.0%	28.0%	36.0%
Other commitments (work, family, school, etc.)	21.7%	17.4%	39.1%	8.7%	13.0%
Knowing participating in a study makes me feel like I matter	12.5%	12.5%	16.7%	20.8%	37.5%
Knowing participating in a study makes me feel better	12.5%	16.7%	12.5%	12.5%	45.8%

I would participate in a vaccine study for monetary compensation…yes, it's important, and it helps science and medicine, and that's all well and good, but I need money.

### Barriers to participation in HIV vaccine research

The participants identified the following barriers to participation most frequently in HIV vaccine trials: misinformation about vaccines and feelings of exclusion from research.

### Misinformation about vaccines

The participants expressed a variety of misinformation about potential side effects of HIV vaccine study products in the focus group, though the majority (74.0%) had heard about an HIV vaccine based on their survey responses. One participant noted,

Whether it's a rational fear or not, just that thought that you would get HIV from something like that…whether that's even medically possible or not, I don't know, but it's still definitely a big fear.

Besides a fear that an HIV vaccine could actually infect a participant with HIV, participants from every focus group were fearful that the vaccine might interact negatively with hormone therapy while they were gender transitioning. One participant explained:

Additionally, for transgender people…I would need to also know how that's going to interact with my hormone replacement therapy, because there's different brands which you can utilize, and certain ones may interact much differently than others. And, I would just need to know a whole lot about it before I even considered it, because that's then injecting, or ingesting, or however you would receive that into your body, in addition to what you're already medically receiving. And, I can't imagine that putting more and more medications in my body would necessarily be beneficial.

Finally, the participants also expressed misinformation about vaccines and biomedical prevention methods. Many either heard from other friends, the internet, or television that vaccines caused autism or were “here to control populations.” Two illustrative quotes are included below:

I think everybody… that gets a flu shot ends up sick. They always end up sick, or something goes wrong right after they get that flu shot. So, that's why I'm so scared of this, with any type of vaccine.

It's a way for them to control minorities. Oh, they get sick. They've got to deal with that. We deal with less of them. That's what I've heard.

### Feelings of exclusion from research

The majority of participants felt excluded from scientific research. They reported in the focus groups that they were either grouped together with MSM or were excluded from research aimed at biological women. They felt the scientific community saw them as MSM, even though many participants consider themselves heterosexual women who have sex with men. Participants also commented on wanting to be seen and treated as a woman first but acknowledged they were different than biological women.

Trans is not gay, trans is not lesbian, trans is not anything but trans. Just like there's a white person, a black person.

Probably gay men might get it more quicker, or something like that, I feel because it's like when it comes to the transgender community, like I feel a lot of gay men take like a lot of credit for a lot of things. And I know that that's not much to do with this topic, but just to specify a little bit on the history, you know, that's what I feel like, and someone else can agree or disagree.

Finally, participants unanimously felt transgender women were often forgotten or overlooked by HIV researchers with much of the attention paid to MSM. As one woman noted:

There's a lot of HIV activism that's targeted toward homosexual men…but also it kind of ignores the experiences of trans women and the fact that they also can be at high risk for much of the same reason that homosexual men are.

## Discussion

Although the community of transgender women is very diverse, they all face significant barriers to enrolling in research studies and yet share similar motivations for participation. These data are consistent with previous research on facilitators and barriers to participation in HIV vaccine and prevention research among MTF transgender women.^18^

It may be important to consider these themes expressed by focus group particpants as we enagage and recruit MSM and transgender individuals for the upcoming international efficacy study of preventive vaccines.^19^

Even though transgender women view HIV vaccines as an important priority, knowledge of this biomedical prevention strategy remains limited. Significant barriers to trial participation exist such as fear of vaccine side effects, misinformation about vaccines and general feelings of exclusion from, discrimination by and being overlooked by the biomedical research community. However, our findings suggest these barriers may be overcome by leveraging the relationships transgender women have with trusted providers, promoting importance of inclusion in the research process, as well as disseminating accurate information via online social networks.

Three key concepts emerged from these data that would be important to emphasize in educational and recruitment materials developed specifically for transgender women, including how participation helps the community, the safety of experimental vaccines, and how much they are reimbursed in cash for their time. Delivery channels for marketing material that emphasize a personal connection, such as a clinician's office, as well as confidentiality, such as Craigslist and Reddit, would likely reach the widest audience.

### Limitations

This study recruited transgender women in the New York City tri-state area who were either engaged with a local CBO or had an online presence via Craiglist or Facebook. Furthermore, we excluded transgender women who had used injection drugs in the past 12 months or were known to be HIV-positive. It should therefore not be considered representative of all transgender women and may underrepresent the opinions of the most at-risk individuals who stand to benefit the most from biomedical preventative measures. Finally, since the collection of these data almost 4 years ago, there have been increased efforts in popular media and the medical community to be more inclusive of transgender women. As a result, our results may be somewhat tempered in today's environment but likely thematically still very relevant. Further research should explore this potentially higher risk population and focus on issues such as stigma and structural barriers to participation in research.

## Conclusions

Given the burden of HIV borne by transgender women, it is an important group to include in HIV vaccine and biomedical prevention studies as well as in the research process itself. Educational and recruitment materials should be developed with input from local communities of transgender women. To promote feelings of inclusion in the research process, transgender women should be included in research activities as distinct from MSM. They should, for example, be represented in Community Advisory Boards and as research staff members of both clinical research sites and community partners who are responsive to the unique needs of the community, to begin to break down barriers and enhance facilitators to participation in HIV biomedical prevention research.

## Abbreviations Used

CBO: community based organizations
MSM: men who have sex with men
